# Pimarane Diterpenoids from the Seeds of *Caesalpinia minax* as PTP1B Inhibitors and Insulin Sensitizers

**DOI:** 10.3390/molecules25204674

**Published:** 2020-10-13

**Authors:** Yunshao Xu, Zheling Feng, Tian Zhang, Peng Lv, Jun Cao, Dan Li, Cheng Peng, Ligen Lin

**Affiliations:** 1 State Key Laboratory of Quality Research in Chinese Medicine, Institute of Chinese Medical Sciences, University of Macau, Macao SAR 999078, China; mb75820@um.edu.mo (Y.X.); yb77508@um.edu.mo (Z.F.); yb67520@um.edu.mo (T.Z.); yb87503@um.edu.mo (P.L.); yb97509@um.edu.mo (J.C.); 2 State Key Laboratory of Southwestern Characteristic Chinese Medicine Resources, School of Pharmacy, Chengdu University of Traditional Chinese Medicine, Chengdu 610075, China; lidan@cdutcm.edu.cn (D.L.); pengchengchengdu@126.com (C.P.)

**Keywords:** pimarane diterpenoids, *Caesalpinia minax*, PTP1B, glucose uptake, C2C12 myotubes

## Abstract

Protein-tyrosine phosphatase 1B (PTP1B) has been considered as a promising target for treating insulin resistance. In searching for naturally occurring PTB1B antagonists, two new pimarane diterpenoids, named 2α-hydroxy-7-oxo-pimara-8(9),15-diene (**1**) and 19-hydroxy-2α-acetoxy-7-oxo-pimara-8(9),15-diene (**2**), were isolated from the seeds of *Caesalpinia minax*. Their structures were determined by extensive analysis of NMR and HR-ESIMS data, and their absolute configurations were determined by electronic circular dichroism (ECD) spectra. Compound **1** was disclosed as a competitive inhibitor of PTP1B with an IC_50_ (the half-maximal inhibitory concentration) value of 19.44 ± 2.39 µM and a *K*i (inhibition constant) value of 13.69 ± 2.72 μM. Moreover, compound **1** dose-dependently promoted insulin-stimulated glucose uptake in C2C12 myotubes through activating insulin signaling pathway. Compound **1** might be further developed as an insulin sensitizer.

## 1. Introduction

Protein tyrosine phosphatases (PTP) are specific, tightly regulated, and critical modulators of cellular signal initiation, transduction and termination [1]. PTP1B dephosphorylates tyrosine residues in insulin receptor (IR) and IR substrate 1 (IRS1), reducing insulin sensitivity and shutting down insulin signaling [1,2]. Additionally, PTP1B dephosphorylates leptin receptor and Janus kinase 2, functioning as a negative regulator of leptin signaling [1]. Thus, PTP1B inhibitors enhance the sensitivities of insulin and leptin signaling and have favorable curing effect for diabetes and obesity [1,2]. Up to now, only two small-molecular PTP1B antagonists, ertiprotafib [3] and trodusquemine [4], have reached clinical trials, but both were failed. Hence, it is urgent to identify potent and selective small-molecular PTP1B inhibitors. Natural products, including fatty acids, phenolics, terpenoids, steroids and alkaloids, provide a great amount of PTP1B inhibitors with structural diversity [5,6].

The seeds of *Caesalpinia minax* Hance (Fabaceae), called “ku-shi-lian” in China, have been traditionally used for healing dysentery, rheumatism and the common cold [7]. Lots of phytochemical investigations have identified more than 150 cassane diterpenoids from the titled species [8,9]. Although cassane diterpenoids are considered as the rearranged products of pimarane precursors through a methyl migration from C-13 to C-14, pimarane diterpenoids were seldom reported from the genus *Caesalpinia*. Till now, only two pimarane diterpenoids, pulcherrin R and tomocinol C, were isolated from *Caesalpinia* species [10,11]. In a search for PTP1B inhibitors, two new pimarane diterpenoids were identified from the seeds of *C. minax* Hance. Herein, the isolation and structure elucidation of new pimarane diterpenoids, as well as their PTP1B inhibitory and insulin-sensitizing activities, are reported.

## 2. Results and Discussions

### 2.1. Structure Elucidation

**Figure 1:**
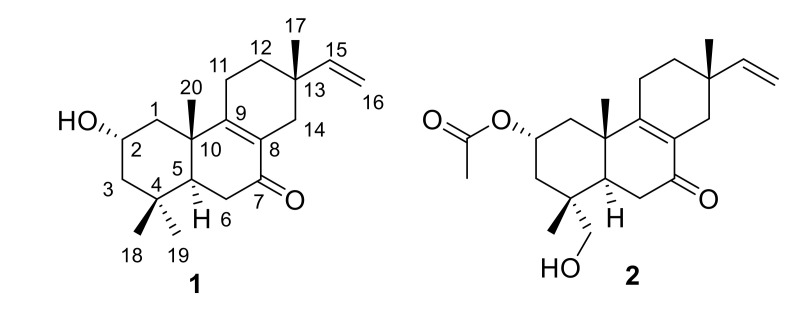
Chemical structures of compounds **1** and **2**.

Compound **1**, a white amorphous powder, possessed a molecular formula of C_20_H_30_O_2_ based on the protonated ion peak at *m*/*z* 303.2326 (calcd. for C_20_H_31_O_2_, 303.2324) in the HRESIMS (high resolution electrospray ionization mass spectrometry), indicating six degrees of unsaturation. The broad infrared (IR) absorption at 3452 cm^−1^ suggested the presence of hydroxy group in the structure of **1**. The ultraviolet (UV) absorption maximum at 245 nm together with the IR absorption at 1742 cm^−1^ indicated the existence of an α,*β*-unsaturated ketone moiety [12]. The ^1^H-NMR (proton nuclear magnetic resonance) spectrum of **1** (Table 1) displayed resonances for four methyls [*δ*_H_ 1.10 (3H, s), 0.97 (3H, s), 0.96 (3H, s) and 0.95 (3H, s)], one oxygenated methine [*δ*_H_ 3.99 (1H, tt, *J* = 11.5, 4.1 Hz)], and three olefinic protons ascribed to a vinyl group [*δ*_H_ 5.74 (1H, dd, *J* = 17.4, 10.8 Hz) and 4.90 (2H, m)]. The ^13^C-NMR (carbon-13 nuclear magnetic resonance) and DEPT-135 (distortionless enhancement by polarization transfer-135) spectra of **1** (Table 1) exhibited 20 resonances attributed to one ketone carbonyl carbon (*δ*_C_ 199.7), four olefinic carbons (*δ*_C_ 164.6, 147.2, 129.1 and 111.0), three quaternary carbons (*δ*_C_ 41.2, 34.8 and 34.3), two methine carbons (*δ*_C_ 65.1 and 49.4), six methylene carbons (*δ*_C_ 50.5, 45.0, 35.3, 33.6, 33.1 and 22.9) and four methyl carbons (*δ*_C_ 32.6, 24.9, 22.4 and 19.5). The above evidence revealed that **1** possesses a pimarane diterpenoid framework, and its ^1^H- and ^13^C-NMR data quite resembled those of isopimara-8,15-dien-7-one [12,13]. The main difference was a methylene group in isopimara-8,15-dien-7-one was replaced by an oxygenated methine (*δ*_H_ 3.99/*δ*_C_ 65.1) in **1**. The HMBC (heteronuclear multiple bond correlation) cross peaks between the oxygenated methine proton (*δ*_H_ 3.99) and C-1 (*δ*_C_ 45.0), C-3 (*δ*_C_ 50.5), C-4 (*δ*_C_ 34.8) and C-10 (*δ*_C_ 41.2) assigned it as C-2, which was further supported by the coupling constant of H-2. Thus, the planar structure of compound **1** was established. The relative configuration of **1** was inferred by ROESY (rotating frame Overhause effect spectroscopy) experiment, indicating the same as pimarane diterpenoids. The NOE (nuclear Overhause effect) correlations between H-2 and H_3_-18 and H_3_-20 indicated that the hydroxy group on C-2 was α-oriented. To identify the absolute configuration of **1**, an electronic circular dichroism (ECD) spectrum was acquired and analyzed. Compound **1** exhibited negative and positive Cotton effects at 250.5 and 322.0 nm, respectively, matching the spectrum for the normal pimarane quite well [14]. Thus, the absolute configuration of **1** was determined as 2*S*, 5*S*, 10*S*, and 13*S*. Accordingly, the structure of **1** was established, and it was named 2α-hydroxy-7-oxo-pimara-8(9),15-diene (Figure 1).

Compound **2** was obtained as a white amorphous powder. Its molecular formula was assigned as C_22_H_32_O_4_ according to the protonated ion peak at *m*/*z* 361.2374 [M + H] ^+^ (calcd. for C_22_H_32_O_4_, 361.2379) in its HRESIMS. The presences of hydroxy and ketone groups in the structure of **2** were inferred by the broad IR absorption at 3442 cm^−1^ and the sharp IR absorption at 1729 cm^−1^, respectively. The ^1^H-NMR spectrum exhibited signals for three methyl group [*δ*_H_ 1.18 (3H, s), 0.95 (3H, s), and 0.94 (3H, s)], one acetoxy group [2.05 (3H, s)], one oxygenated methine [*δ*_H_ 5.16 (1H, tt, *J* = 11.7, 4.3 Hz)], one oxygenated methylene [*δ*_H_ 3.43 (1H, d, *J* = 10.9 Hz) and 3.15 (1H, d, *J* = 10.9 Hz)], and three olefinic protons ascribed to a vinyl group [*δ*_H_ 5.73 (1H, dd, *J* = 17.4, 10.8 Hz) and 4.90 (2H, m)] (Table 1). The ^13^C-NMR spectrum of **2** exhibited 22 carbon resonances (Table 1). The ^1^H- and ^13^C-NMR data for compound **2** were quite similar to those of compound **1**. After careful analysis of 2D NMR, the hydroxy group at C-2 in **1** was replaced by an acetoxy group in **2**. In the HMBC spectrum, the correlations from the oxygenated methylene protons to C-2 (*δ*_C_ 68.4), C-3 (*δ*_C_ 40.0), C-4 (*δ*_C_ 39.0) and C-5 (*δ*_C_ 42.3), indicated that one of the methyl groups on C-4 was replaced by a hydroxymethyl group in **2**. Next, a ROSEY experiment was carried out to elucidate the relative configuration of **2**. The NOE correlations between H-2 and H_3_-18 and H_3_-20, as well as between H-5 and H_2_-19, indicated that the hydroxy group was attached to C-19 and the acetoxy group on C-2 was α-oriented. The ECD spectrum indicated the absolute configuration of **2** to be the same as that of **1**, assigning as 2*S*, 3*S*, 5*S*, 10*S*, and 13*S*. Therefore, the structure of **2** was established, named 19-hydroxy-2α-acetoxy-7-oxo-pimara-8(9),15-diene (Figure 1).

### 2.2. PTP1B Inhibitory Activity

**Figure 2:**
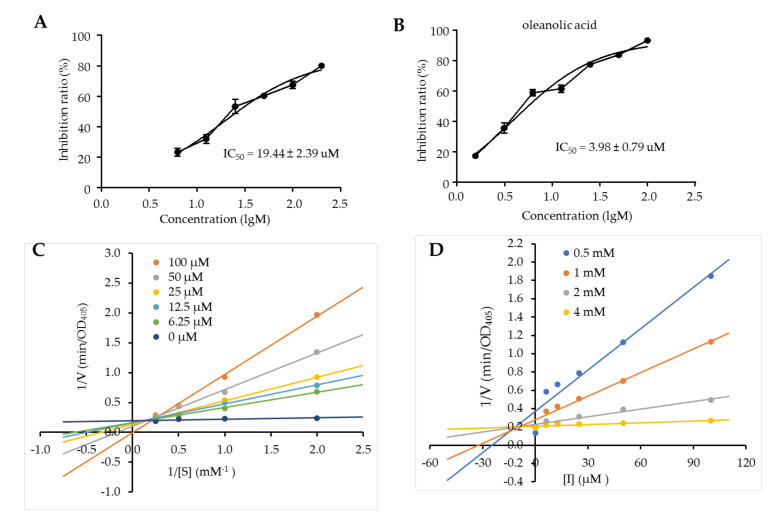
Protein-tyrosine phosphatase 1B (PTP1B) inhibitory activity and kinetic analysis for compound **1**. Concentration—PTP1B inhibition ratio curves of compound **1** (**A**) and the positive control oleanolic acid (**B**). (**C**) Lineweaver-Burk plot for the inhibition of PTP1B by compound **1**. (**D**) Dixon plot for the inhibition of the PTP1B by compound **1**. Data are expressed as mean ± SD, *n* = 4.

Compounds **1** and **2** were assayed for their PTP1B inhibitory activity using 4-nitrophenyl phosphate disodium salt (pNPP) as the dephosphorylating substrate. pNPP is dephosphorylated to yield the product *p*-nitrophenol, which could be monitored at an absorbance of 405 nm and the slope of the initial rate of the kinetic curve in each well was applied to determine the activity of PTP1B [15]. Oleanolic acid, a well-documented PTP1B inhibitor, was used as a positive control [15]. Compounds **1** and **2**, as well as oleanolic acid, were dissolved in dimethyl sulfoxide (DMSO) to make 10 mM stock solutions, which were further diluted in reaction buffer or cell culture medium to the corresponding concentrations. Under the concentration of 200 μM, compound **1** and oleanolic acid reduced PTP1B activity at the rate of 76.6% and 93.4%, respectively, but not compound **2** (6.5%). Thus, compound **1** was selected for further studies. Compound **1** dose-dependently inhibited PTP1B activity with an IC_50_ (the half-maximal inhibitory concentration) value of 19.44 ± 2.39 μM (Figure 2A), which was comparable to that of oleanolic acid (IC_50_ = 3.98 ± 0.98 μM) (Figure 2B).

To further investigate the PTP1B enzyme inhibition type and inhibition constant (*K*i) of compound **1**, Lineweaver-Burk and Dixon plots were analyzed with different concentrations of compound **1** (0, 6.25, 12.5, 25, 50 and 100 μM) and pNPP (0.5, 1, 2 and 4 mM), respectively [16,17]. Competitive inhibitors have the same y-intercept but different slopes and x-intercepts; non-competitive inhibition produces plots with the same x-intercept but different slopes and y-intercepts; and uncompetitive inhibition causes different intercepts on both the y- and x-axes. The Lineweaver–Burk (Figure 2C) and Dixon plots (Figure 2D) of compound **1** intersected at the y-side, revealing a competitive inhibition mode. Compound **1** might directly interact with the active binding sites of the PTP1B enzyme. The Dixon plot has been widely recruited to determine the *K*i for an enzyme-inhibitor complex [17]. Herein, the *K*i value of **1** was calculated to be 13.69 ± 2.72 μM, indicating compound **1** was a potent PTP1B inhibitor.

### 2.3. Glucose Uptake Stimulatory Property on C2C12 Myotubes

**Figure 3:**
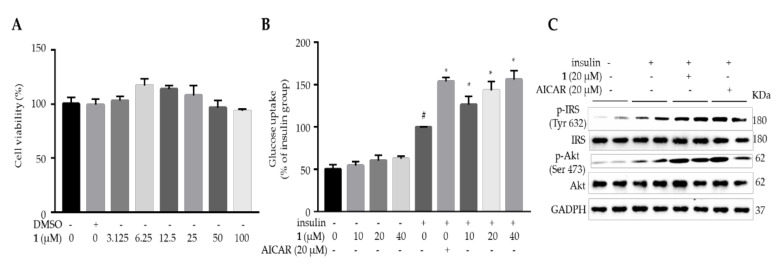
Compound **1** enhanced insulin-stimulated glucose uptake on C2C12 myotubes through activating insulin signaling pathway. (**A**) Cytotoxicity of compound **1** on C2C12 myotubes when treated for 24 h by MTT assay. (**B**) Compound **1** increased insulin-stimulated glucose uptake on C2C12 myotubes. AICAR was used as a positive control. (**C**) Compound **1** activated the insulin signaling pathway in C2C12 myotubes. The expressions of *p*-IRS-1, IRS-1, *p*-Akt, Akt, and GAPDH were analyzed by Western blots. “+” means presence, and “−” means absence. Data are expressed as mean ± SD, *n* = 6. # *p* < 0.0001, control vs. insulin; * *p* < 0.001, compound **1** or AICAR vs. insulin.

Mouse C2C12 myotubes have been widely used as an *in vitro* model to mimic skeletal muscle [18,19]. The fully differentiated C2C12 myotubes uptake glucose from culture medium upon insulin stimulation, which has been widely used to screen and validate insulin sensitizer [18]. Herein, the glucose uptake stimulatory property of compound **1** was evaluated on C2C12 myotubes. 5-Aminoimidazole-4-carboxamide ribonucleotide (AICAR, 20 μM), an activator of AMP-activated protein kinase (AMPK), was used as a positive control. Firstly, the non-cytotoxic concentrations of compound **1** (3.125, 6.25, 12.5, 25, 50 and 100 μM) on C2C12 myotubes were evaluated by the MTT [3-(4,5-dimethylthiazol-2-yl)-2,5-diphenyltetrazolium bromide] assay, to determine the maximum safe dosage. Compound **1** didn’t show obvious cytotoxicity up to 50 μM when treated for 24 h (Figure 3A). Next, insulin-stimulated glucose uptake on C2C12 myotubes was evaluated to determine the insulin-sensitizing effect of compound **1** (10, 20 and 40 μM). Compound **1** didn’t obviously affect glucose uptake without insulin stimulation (Figure 3B). As expected, insulin dramatically enhanced glucose uptake, and compound **1** further increased insulin-stimulated glucose uptake on C2C12 myotubes, in a dose-dependent manner (Figure 3B). At the concentration of 40 μM, compound **1** increased insulin-stimulated glucose uptake by 56.0%, compared with the insulin-treated cells. Upon insulin stimulation, its receptor is autophosphorylated on tyrosine residues, which in turn phosphorylates and recruits IRS1 (Tyr 632). Tyrosine phosphorylated IRS1 further activates the phosphatidylinositol 3-kinase (PI3K) and protein kinase B (Akt, Ser 473), leading to cell membrane translocation of glucose transporter type 4 (GLUT4) to uptake glucose [20]. Thus, the key regulators of insulin signaling pathway in compound **1**-treated C2C12 myotubes were evaluated by using Western blots. Compound **1** treatment (20 μM) increased insulin-stimulated phosphorylation of IRS1 and Akt, when compared with those of insulin-treated cells (Figure 3C). Thus, compound **1** improved insulin sensitivity on C2C12 myotubes.

## 3. Materials and Methods

### 3.1. General Experimental Procedures

Optical rotation data were recorded on an Autopol VI polarimeter (Rudolph Research Analytical, Hackettstown, NJ, USA). UV absorption was monitored with a Varian CARY 50 spectrophotometer (Agilent Technologies, Clara, CA USA). IR spectra were obtained on a PerkinElmer spectrum-100 FTIR spectrometer (PerkinElmer, Waltham, MA, USA) using KBr disks. Electronic circular dichroism (ECD) spectra were measured on a Jasco J-180 spectrophotometer (Jasco, Tokyo, Japan). NMR spectra were recorded on a Bruker Avance-600 NMR spectrometer (Bruker, Fällanden, Switzerland). The chemical shift *δ* values were presented in ppm, and the coupling constants (*J*) were given in Hz. HRESIMS spectra were recorded on an LTQ-Orbitrap XL spectrometer (ThermoFisher Scientific, Bremen, Germany). Silica gel (300‒400 mesh, Qingdao Haiyang Chemical Co., Ltd., Qingdao, China) and MCI gel (CHP20P, 75‒150 μm, Mitsubishi Chemical Industries Ltd., Tokyo, Japan) were used for column chromatography (CC). All solvents were analytical grade (TianJing Chemical Plant, Tianjing, China). Precoated silica gel GF254 plates (Qingdao Haiyang Chemical Co., Ltd.) were used for TLC. TLC spots were viewed at 254 nm and visualized by spraying with 5% H_2_SO_4_ in EtOH. Preparative HPLC was performed on a Shimadzu LC-20AP instrument (Shimadzu, Kyoto, Japan) with a SPD-M20A PDA detector (Shimadzu, Kyoto, Japan), using a C18 column (19 × 250 mm, 5 μm, Waters, SunFire^TM^, Milford, MA, USA) and a gradient solvent system comprised of H_2_O and CH_3_CN at a flow rate of 10 mL/min.

### 3.2. Plant Material

The seeds of *C. minax* were collected from Zhaoping County, Guangxi Zhuang Autonomous Region, People’s Republic of China, in October 2018, and identified by Professor Changqiang Ke from Shanghai Institute of Materia Medica, Chinese Academy of Sciences (Shanghai, China). A voucher was deposited at the herbarium of the Institute of Chinese Medical Sciences, University of Macau (No. 20181003).

### 3.3. Extraction, Isolation and Characterization of Compounds

The air-dried seeds of *C. minax* (15 kg) were ground and extracted with 95% ethanol (45 L × 3 times, each 7 days). The pooled extracts were concentrated under reduced pressure to yield a residue (1.58 kg), which was then suspended in hot water (5 L) and extracted with petroleum ether (PE, 3 L × 3 times), chloroform (3 L × 3 times), and EtOAc (3 L × 3 times), successively, yielding a PE (586 g), a chloroform (86 g) and an EtOAc (15 g) fraction. The chloroform fraction (85 g) was separated by CC over silica gel eluted with PE/acetone (20:1, 15:1, 7:1, 4:1, 3:1, 2:1, and 1:1, *v*/*v*), giving 10 fractions (A–J). Fraction C (3.9 g) was subjected to an MCI gel column eluted with CH_3_OH/H_2_O (0:1 to 0:1) to obtain five subfractions (C1 to C5). Fraction C4 (450 mg) was purified by preparative HPLC, eluting with CH_3_CN/H_2_O (80‒83%, *v*/*v*), to obtain compound **1** (3.7 mg, Supplementary Materials Figure S11). Fraction D (1.9 g) was subjected to CC over MCI gel, eluted with CH_3_OH/H_2_O (0:1 to1:0), to yield ten subfractions (D1 to D10). Fraction D8 (128 mg) was separated by preparative HPLC, eluting with CH_3_CN/H_2_O (80‒100%, *v*/*v*), to obtain compound **2** (7.8 mg, Supplementary Materials Figure S21).

#### 3.3.1. α-Hydroxy-7-oxo-pimara-8(9),15-diene (**1**)

White amorphous powder; [α]^25^_D_ +1.2 (*c* 0.37, CH_3_OH); UV (CH_3_OH) *λ*_max_ (log *ε*) 200.0 (0.54), 245.0 (0.54) nm; ECD (CH_3_OH, nm) *λ*_max_ (Δ*ε*) 204.0 (+2.2), 250.5 (−5.7), 322.0 (+1.2); IR *ν*_max_ (KBr) 3452 (strong, broad), 2918, 2854, 1742, 1682, 1393, 1203, 1134, 1023 cm^−1^; ^1^H- and ^13^C-NMR data (see Table 1); HRESIMS *m*/*z* 303.2326 [M + H] ^+^ (calcd for C_20_H_31_O_2_, 303.2324).

#### 3.3.2. 19-Hydroxy-2α-acetoxy-7-oxo-pimara-8(9),15-diene (**2**)

White amorphous powder; [α]^25^_D_ −4.4 (*c* 0.78, CH_3_OH); UV (CH_3_OH) *λ*_max_ (log *ε*) 194.9 (0.53), 250.1 (0.40) nm; ECD (CH_3_OH, nm) *λ*_max_ (Δ*ε*) 220.0 (+4.7), 253.5 (−5.9), 327.0 (+1.5); IR *ν*_max_ (KBr) 3442 (strong, broad), 2930, 2871, 1729, 1658, 1378, 1243, 1030 cm^−^^1^; ^1^H- and ^13^C-NMR data (see Table 1); HRESIMS *m*/*z* 361.2374 [M + H] ^+^ (calcd for C_22_H_33_O_4_, 361.2379).

### 3.4. PTP1B Assay

PTP1B enzymatic reaction was performed as described previously [15,21]. The PTP1B enzymatic activity was measured in 100 μL reaction buffer [1 mM bovine serum albumin (BSA), 50 mM 3-(*N*-morpholino)-propanesulfonic acid, 1 mM dithiothreitol, and 1 mM ethylenediaminetetraacetic acid (EDTA), pH 6.5] containing 15 nM human recombinant PTP1B enzyme (Sigma-Aldrich, St Louis, MO USA) and 10 mM pNPP (Sigma-Aldrich, St Louis, MO, USA), in 96-well plates. Different concentrations of compound **1** and oleanolic acid were added in the designated wells, and the plate was pre-incubated at 37 °C for 30 min. Subsequently, pNPP was added to each well, and the plate was incubated at 37 °C for 45 min. 50 μL NaOH (3 M) was added to each well to stop reaction. pNPP was dephosphorylated to yield the product *p*-nitrophenol, which could be monitored at an absorbance of 405 nm with a SpectraMax M5 microplate reader (Molecular Devices, San Jose, CA, USA), and the slope of the initial rate of the kinetic curve in each well was applied to determine the activity of PTP1B. The wells with only DMSO and pNPP were considered as blank control; the wells with DMSO, pNPP and enzyme were considered as negative control. The PTP1B inhibitory ratio was calculated as: (A_negative_ − A_sample_)/(A_negative_ − A_blank_) × 100%. A_negative_, A_sample_ and A_blank_ were absorbance for negative control, sample and blank control, respectively. The IC_50_ value was calculated with Graphpad Prism 6.0 software (GraphPad, San Diego, CA, USA). The PTP1B enzyme kinetic assay was performed as described above. The absorbance was recorded every 5 min for a half hour. The Dixon plot experiment was performed with various concentrations of *p*-NPP, and the Lineweaver-Burk plot experiment was performed with various concentrations of compound **1**. The *K*i value was obtained from the interpretation of Dixon plots.

### 3.5. Cell Culture

C2C12 myoblasts were obtained from American Type Culture Collection (Manassas, VA, USA) and maintained in Dulbecco’s modified Eagle medium (DMEM, Thermo Fisher, Waltham, MA, USA) with 10% fetal bovine serum (FBS, Thermo Fisher, Waltham, MA, USA) and 1% penicillin-streptomycin (P/S), in a 5% CO_2_ incubator at 37 °C. When reach 70‒80% confluence, cells were incubated with fresh medium containing 2% heat-inactivated horse serum (HS, Thermo Fisher, Waltham, MA, USA) and 1% P/S for 4 days, to differentiate into myobutes. Media were changed every day.

### 3.6. Cell Viability

MTT assay was performed to determine cell viability as described previously [18,19]. The fully differentiated C2C12 myotubes were treated with different concentrations of compounds for 24 h. DMSO was used as a blank control. Then cells were incubated with DMEM containing 1 mg/mL MTT for additional 4 h. 100 μL DMSO was added to each well to dissolve the formazan crystals. The absorbance at 570 nm was recorded by a SpectraMax M5 microplate reader. The cell viability was calculated as following: (As − A0)/(Ac − A0) × 100%, where As, Ac and A0 were the absorptions of test sample, negative control (DMSO) and blank control, respectively.

### 3.7. Insulin Stimulated Glucose Uptake

Glucose uptake was performed as described previously [22]. In brief, C2C12 myotubes were treated with different concentrations of compound **1** for 24 h. After washed with Krebs-Ringer’s phosphate (KRP) buffer (137 mM NaCl, 20 mM HEPES, 4.7 mM KCl, 1.2 mM KH_2_PO_4_, 1.2 mM MgSO_4_, 2 mM pyruvate and 2.5 mM CaCl_2_, pH 7.4) twice, the cells were incubated for 3 h in KRP buffer containing 0.2% BSA. Subsequently, the cells were stimulated insulin (0.1 μM in KRP buffer) for 30 min. After washed with KRP buffer once, the cells were incubated in 2-NBDG solution [2-(*N*-(7-nitrobenz-2-oxa-1, 3-diazol-4-yl) amino)-2-deoxyglucose (Sigma-Aldrich, St Louis, MO, USA), 100 μM in KRP buffer] for 30 min. The intracellular content of 2-NBDG was recorded by a fluorescence spectrometer (excitation wavelength: 475 nm; emission wavelength: 550 nm, Molecular Devices, San Jose, CA, USA). The fluorescence intensity was further normalized by protein content.

### 3.8. Western Blot Analysis

Western blot analysis was performed as described previously [23,24]. In brief, protein concentration of sample was determined by using the BCA (bicinchoninic acid) protein assay kit (Thermo Fisher, Waltham, MA, USA). The same amount of proteins (30 μg) were separated by SDS-PAGE (sodium dodecyl sulfate-polyacrylamide gel electrophoresis) and transferred to polyvinylidene fluoride (PVDF) membranes (Bio-Rad, Hercules, CA, USA). Then, the membrane was blocked in TBST buffer (100 mM NaCl, 10 mM Tris-HCl, pH 7.5 and 0.1% Tween-20) with 5% nonfat milk for 1 h at room temperature. After washed with TBST twice, the membrane was incubated with specific primary antibodies overnight at 4 °C. After washing with TBST three times, the membrane was incubated in TBST containing a horseradish peroxidase-conjugated secondary antibody for 2 h at room temperature. The immune-blotting signals were developed using a SuperSignal West Femto Maximum Sensitivity Substrate kit (Thermo Fisher, Waltham, MA, USA) and visualized using the ChemiDoc MP Imaging System. Antibodies against *p*-Akt (Ser 473, cat. no. sc-7985), Akt (cat. no. sc-8312), *p*-IRS-1 (Tyr 632, cat. no. sc-17196), IRS-1 (cat. no. sc-8038), and GAPDH (cat. no. sc-25778) were purchased from Santa Cruz Biotechnology (Santa Cruz, CA, USA).

### 3.9. Statistical Analysis

All data were expressed as mean ± SD (standard deviation) based on at least three independent experiments and analyzed by Graphpad Prism 6 (GraphPad Software, San Diego, CA, USA, https://www.graphpad.com/scientific-software/prism/). One-way ANOVA (analysis of variance) was used for statistical comparison, and *P*-values less than 0.05 were considered statistically significant.

## 4. Conclusions

In summary, the isolation and structure elucidation of two pimarane diterpenoids from *C. minax*, 2α-hydroxy-7-oxo-pimara-8(9),15-diene (**1**) and 19-hydroxy-2α-acetoxy-7-oxo-pimara-8(9),15-diene (**2**), were described in the current study, as well as their PTP1B inhibitory and insulin-sensitizing effects. 2α-Hydroxy-7-oxo-pimara-8(9),15-diene(**1**) potentially inhibits PTP1B enzyme with an IC_50_ value of 13.69 ± 2.72 μM. The enzyme kinetic study indicated that **1** is a competitive inhibitor of PTP1B with a *K*i value of 13.69 ± 2.72 μM. In addition, compound **1** possesses a 2-NBDG uptake enhancing property in insulin-stimulated C2C12 myotubes. The results suggest that pimarane diterpenoids from *C. minax* may be the potential natural products for the development of insulin sensitizers.

## Figures and Tables

**Table 1:** ^1^H- and ^13^C-NMR Spectroscopic Data (CDCl_3_) for Compounds **1** and **2** (*δ*_H_ in ppm, *J* in Hz).

Position	1		2	
	*δ*_H_ (*J* in Hz) *^a^*	*δ*_C_ *^b^*	*δ*_H_ (*J* in Hz)	*δ*_C_
1	α 2.21, m	45.0	α 2.21, m	40.8
	*β* 1.21, m		*β* 1.32, t (11.7)	
2	3.99, tt (11.5, 4.1)	65.1	5.16, tt (11.7, 4.3)	68.4
3	α 1.85, ddd (12.5, 4.1, 2.2)	50.5	α 1.69, m	40.0
	*β* 1.20, m		*β* 1.63, m	
4	‒	34.8	‒	39.0
5	1.71, m	49.4	2.13, m	42.3
6	α 2.53, m	35.3	α 2.45, dd (17.6, 4.0)	34.9
	*β* 2.36, m		*β* 2.35, m	
7	‒	199.7	‒	199.1
8	‒	129.1	‒	129.3
9	‒	164.6	‒	164.3
10	‒	41.2	‒	40.8
11	α 2.31, m	22.9	α 2.25, m	22.9
	*β* 2.25, m		*β* 2.21, m	
12	1.44‒1.52, m (2H)	33.1	1.45‒1.49, m (2H)	33.5
13	‒	33.6	‒	34.3
14	α 2.34, m	33.6	α 2.32, m	33.0
	*β* 1.99, m		*β* 1.97, m	
15	5.74, dd (17.4, 10.8)	147.2	5.73, dd (17.4, 10.8)	147.1
16	4.90, m (2H)	111.0	4.90, m (2H)	111.0
17	0.95, s	24.9	0.95, s	24.9
18	0.96, s	32.6	3.43, 3.15, d (10.9)	70.1
19	0.97, s	22.4	0.94, s	18.2
20	1.10, s	19.5	1.18, s	19.8
2-*O*COCH_3_	‒	‒	‒	170.7
2-*O*COCH_3_	‒	‒	2.05, s	21.6

*^a^* Data measured at 600 MHz. *^b^* Data measured at 150 MHz.

## Supplementary Materials

The following are available online. NMR, IR HRESIMS, ECD and UV spectroscopic data of isolated compounds (**1** and **2**).
