# Supplementary material for: Pimarane Diterpenoids from the Seeds of Caesalpinia minax as PTP1B Inhibitors and Insulin Sensitizers
Source: Molecules. 2020 Oct 13;25(20):4674. doi: 10.3390/molecules25204674 (PMC7587383; doi:10.3390/molecules25204674)
Supplement: Supplementary file 1 [file molecules-25-04674-s001.pdf]

Supporting information

Pimarane Diterpenoids from the Seeds of *Caesalpinia minax* as PTP1B Inhibitors and Insulin Sensitizers

Yunshao Xu [1,†], Zheling Feng [1,†], Tian Zhang [1], Peng Lv [1], Jun Cao [1], Dan Li [2], Cheng Peng [2] and Ligen Lin [1,2,*]

[1] State Key Laboratory of Quality Research in Chinese Medicine, Institute of Chinese Medical Sciences, University of Macau, Macao SAR 999078, China; mb75820@um.edu.mo (Y.X.); yb77508@um.edu.mo (Z.F.); yb67520@um.edu.mo (T.Z.); yb87503@um.edu.mo (P.L.); yb97509@um.edu.mo (J.C.)

[2] State Key Laboratory of Southwestern Characteristic Chinese Medicine Resources, School of Pharmacy, Chengdu University of Traditional Chinese Medicine, Chengdu 610075, China; lidan@cdutcm.edu.cn (D.L.); pengchengchengdu@126.com (C.P.)

* Correspondence: ligenl@um.edu.mo; Tel.: +853-8822-8041

† These authors contributed equally to this work.

Received: 15 September 2020; Accepted: 12 October 2020; Published: 13 October 2020

Fig. S1. ^{1}H NMR (600 MHz, $CDCl_3$) spectrum of 2α-hydroxy-7-oxo-pimara-8(9),15-diene (**1**).

Fig. S2. ^{13}C NMR (150MHz, $CDCl_3$) spectrum of 2α-hydroxy-7-oxo-pimara-8(9),15-diene (**1**).

Fig. S3. DEPT-135 spectrum of 2α-hydroxy-7-oxo-pimara-8(9),15-diene (**1**).

Fig. S4. HSQC spectrum of 2α-hydroxy-7-oxo-pimara-8(9),15-diene (**1**).

Fig. S5. HMBC spectrum of 2α-hydroxy-7-oxo-pimara-8(9),15-diene (**1**).

Fig. S6. ROESY spectrum of 2α-hydroxy-7-oxo-pimara-8(9),15-diene (**1**).

Fig. S7. IR spectrum (KBr disc) of 2α-hydroxy-7-oxo-pimara-8(9),15-diene (**1**).

Fig. S8. HR-ESIMS spectrum of 2α-hydroxy-7-oxo-pimara-8(9),15-diene (**1**) in CH_3OH.

Fig. S9. ECD spectrum of 2α-hydroxy-7-oxo-pimara-8(9),15-diene (**1**) in CH_3OH.

Fig. S10. UV spectrum of 2α-hydroxy-7-oxo-pimara-8(9),15-diene (**1**) in CH_3OH.

Fig. S11. Preparative HPLC chromatogram for 2α-hydroxy-7-oxo-pimara-8(9),15-diene (**1**).

Fig. S12. ^{1}H NMR (600 MHz, $CDCl_3$) spectrum of 19-hydroxy-2α-acetoxy-7-oxo-pimara-8(9),15-diene (**2**).

Fig. S13. ^{13}C NMR (150 MHz, $CDCl_3$) spectrum of 19-hydroxy-2α-acetoxy-7-oxo-pimara-8(9),15-diene (**2**).

Fig. S14. DEPT-135 spectrum of 19-hydroxy-2α-acetoxy-7-oxo-pimara-8(9),15-diene (**2**).

Fig. S15. HMBC spectrum of 19-hydroxy-2α-acetoxy-7-oxo-pimara-8(9),15-diene (**2**).

Fig. S16. ROESY spectrum of 19-hydroxy-2α-acetoxy-7-oxo-pimara-8(9),15-diene (**2**).

Fig. S17. IR spectrum (KBr disc) of 19-hydroxy-2α-acetoxy-7-oxo-pimara-8(9),15-diene (**2**).

Fig. S18. HR-ESIMS spectrum of 19-hydroxy-2α-acetoxy-7-oxo-pimara-8(9),15-diene (**2**) in CH_3OH.

Fig. S19. ECD spectrum of 19-hydroxy-2α-acetoxy-7-oxo-pimara-8(9),15-diene (**2**) in CH_3OH.

Fig. S20. UV spectrum of 19-hydroxy-2α-acetoxy-7-oxo-pimara-8(9),15-diene (**2**) in CH_3OH.

Fig. S21. Preparative HPLC chromatogram for 19-hydroxy-2α-acetoxy-7-oxo-pimara-8(9),15-diene (**2**).

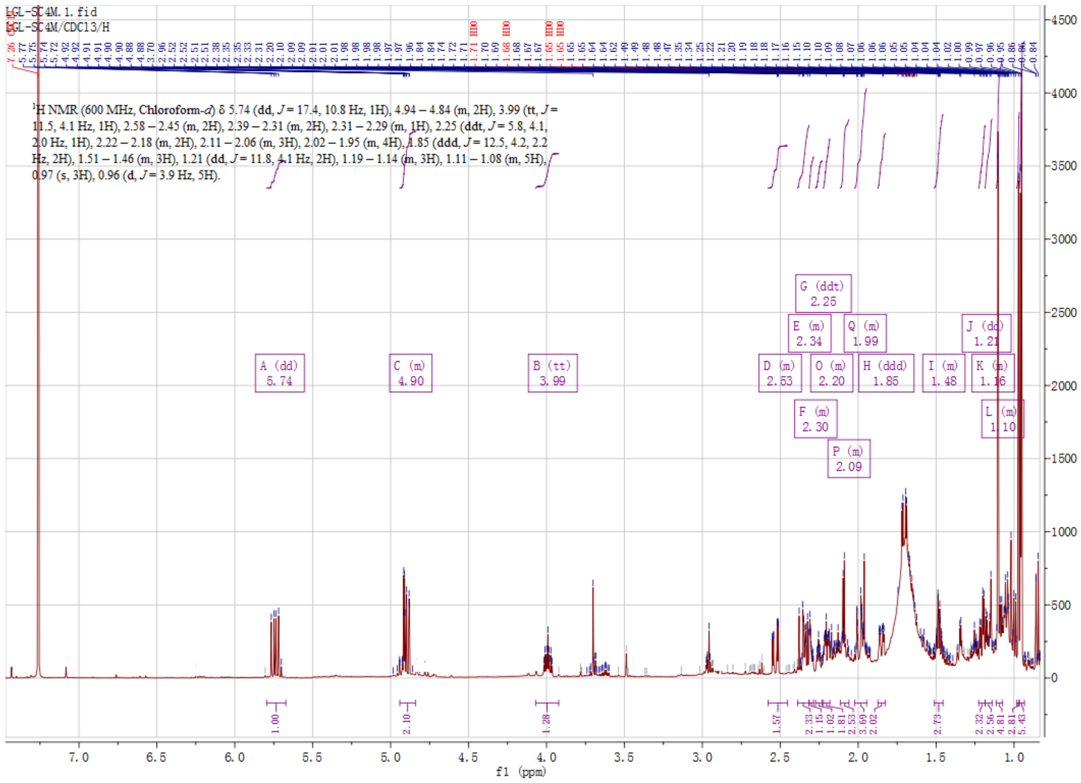

Fig. S1. ^{1}H NMR (600 MHz, $CDCl_3$) spectrum of 2*α*-hydroxy-7-oxo-pimara-8(9),15-diene (**1**).

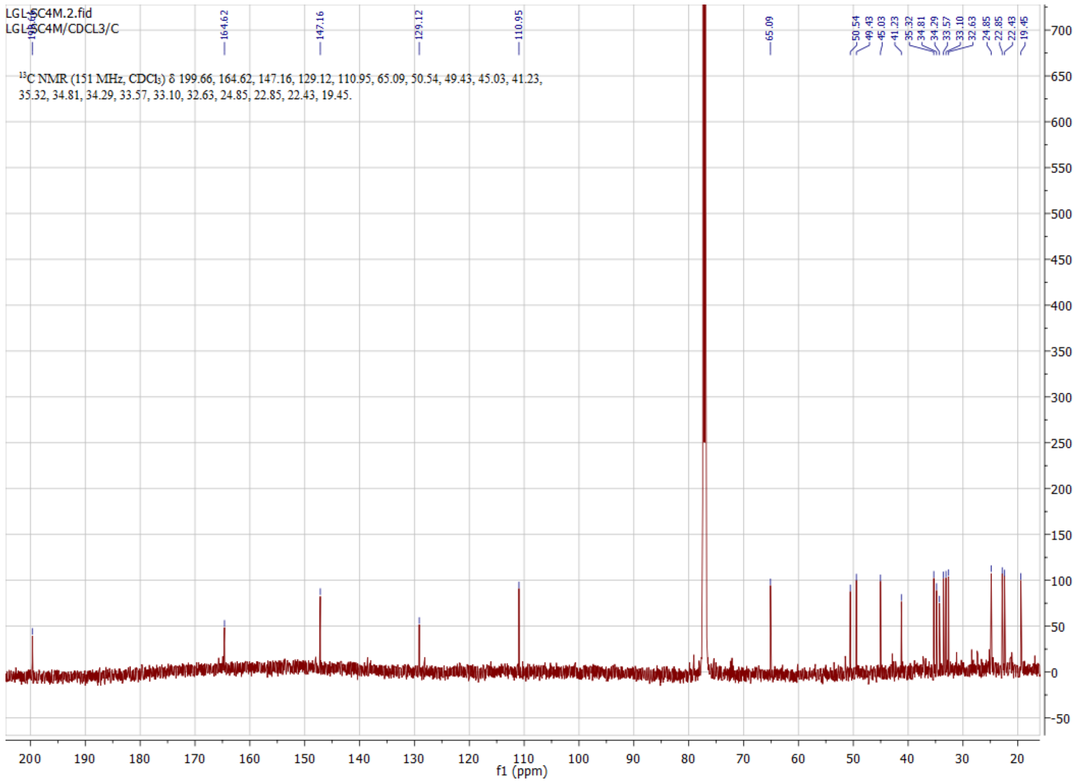

Fig. S2. ^{13}C NMR (150MHz, $CDCl_3$) spectrum of 2*α*-hydroxy-7-oxo-pimara-8(9),15-diene (**1**).

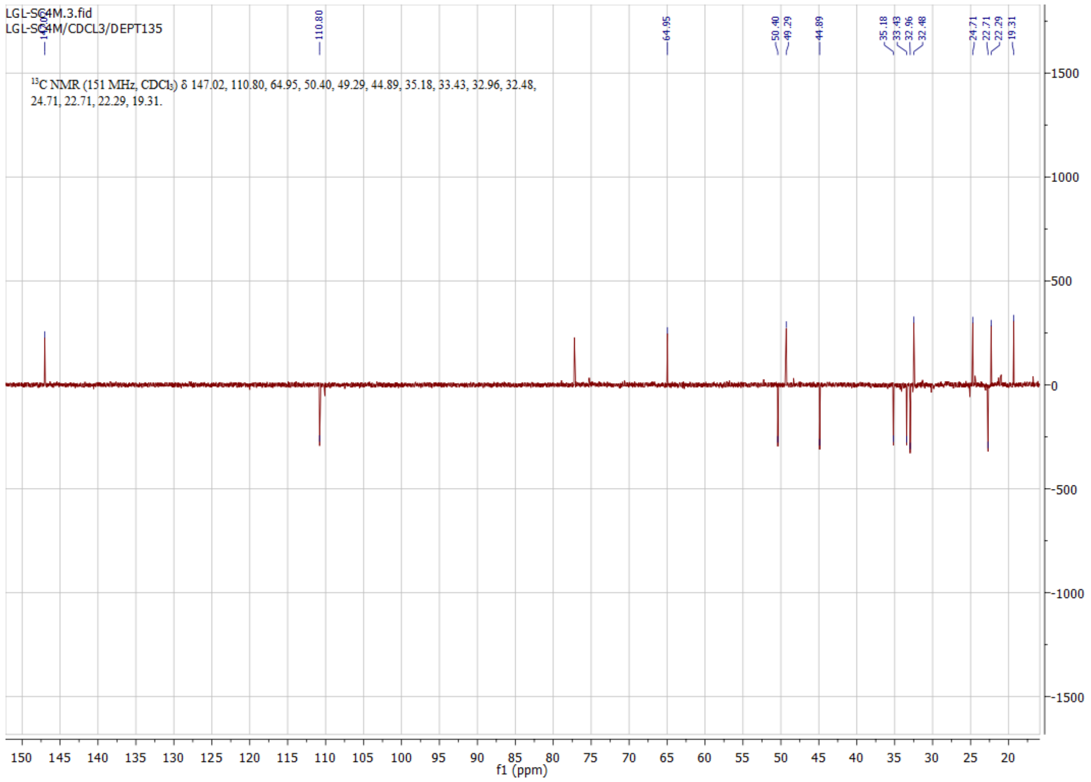

Fig. S3. DEPT-135 spectrum of 2α-hydroxy-7-oxo-pimara-8(9),15-diene (**1**).

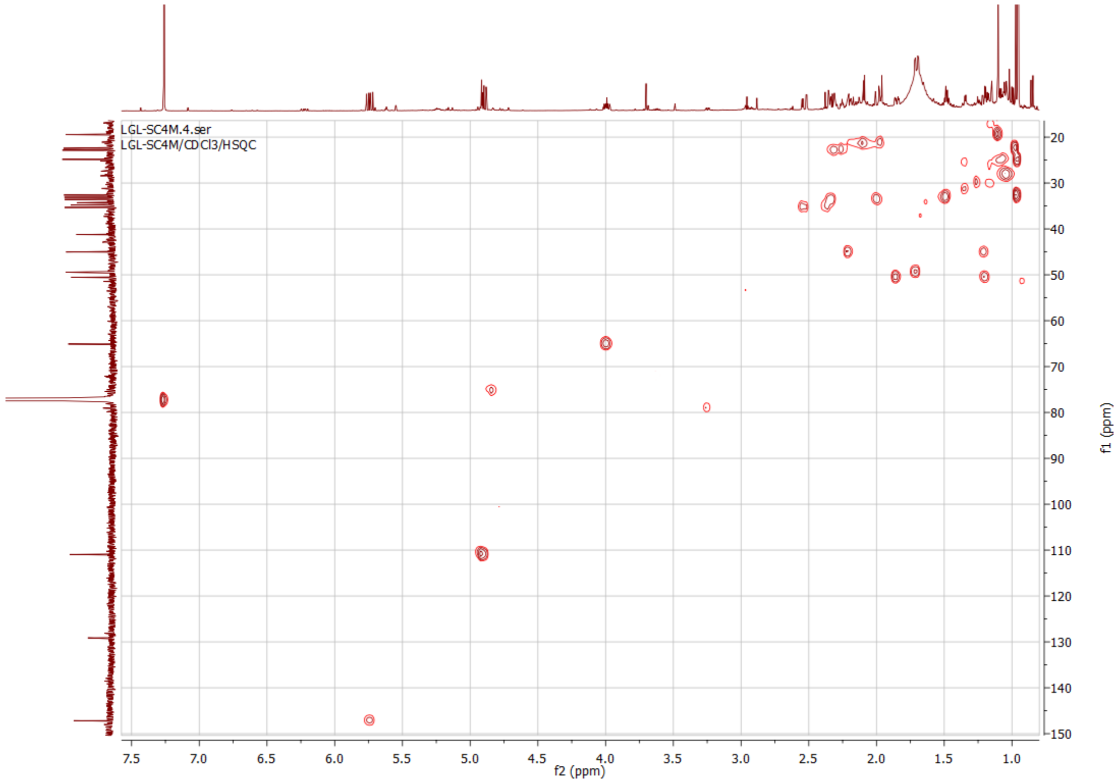

Fig. S4. HSQC spectrum of 2α-hydroxy-7-oxo-pimara-8(9),15-diene (**1**).

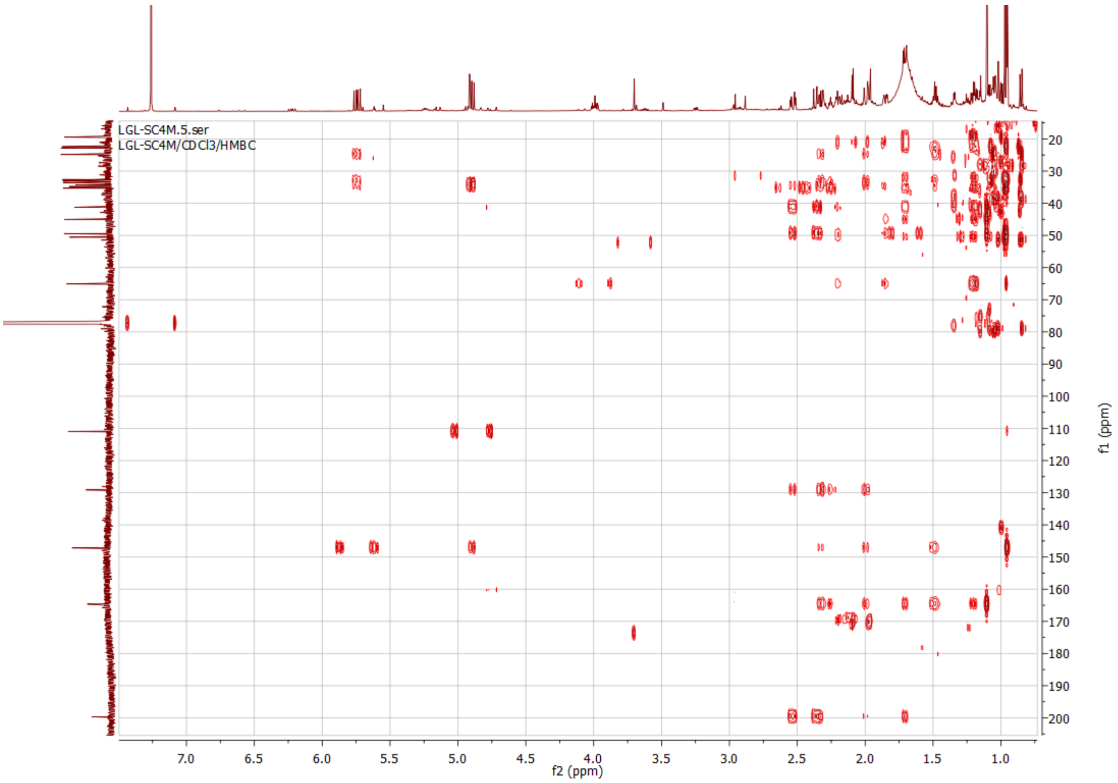

Fig. S5. HMBC spectrum of 2α-hydroxy-7-oxo-pimara-8(9),15-diene (**1**).

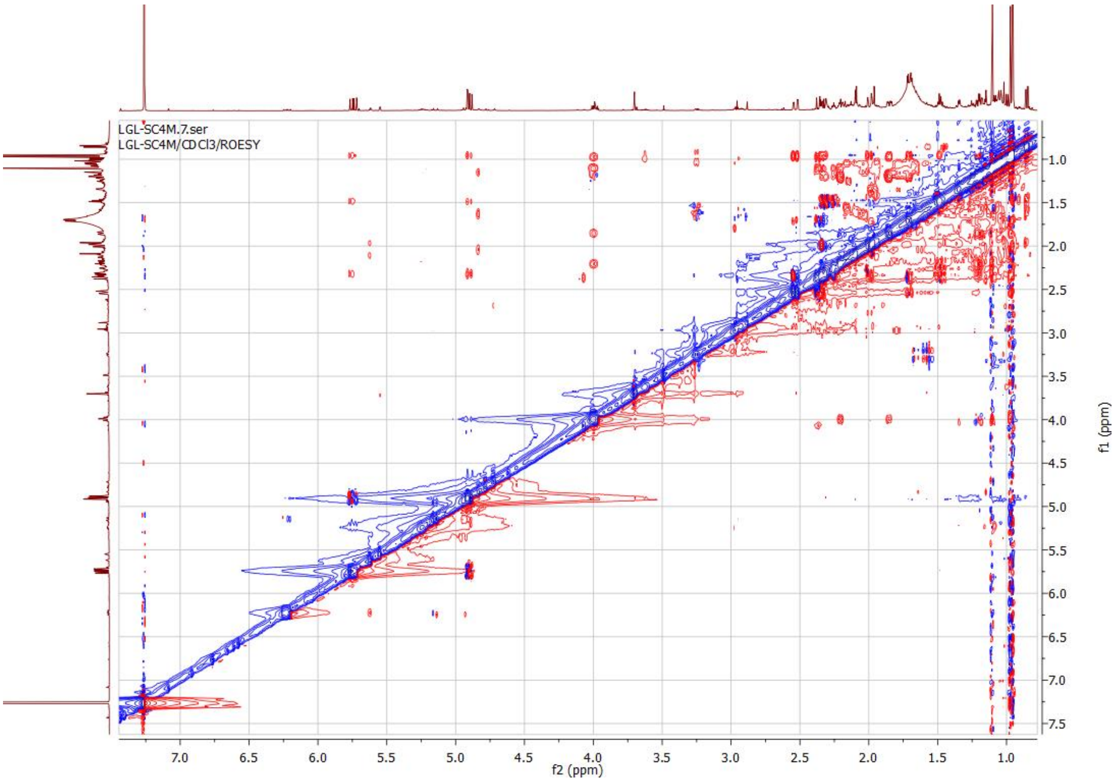

Fig. S6. ROESY spectrum of 2α-hydroxy-7-oxo-pimara-8(9),15-diene (**1**).

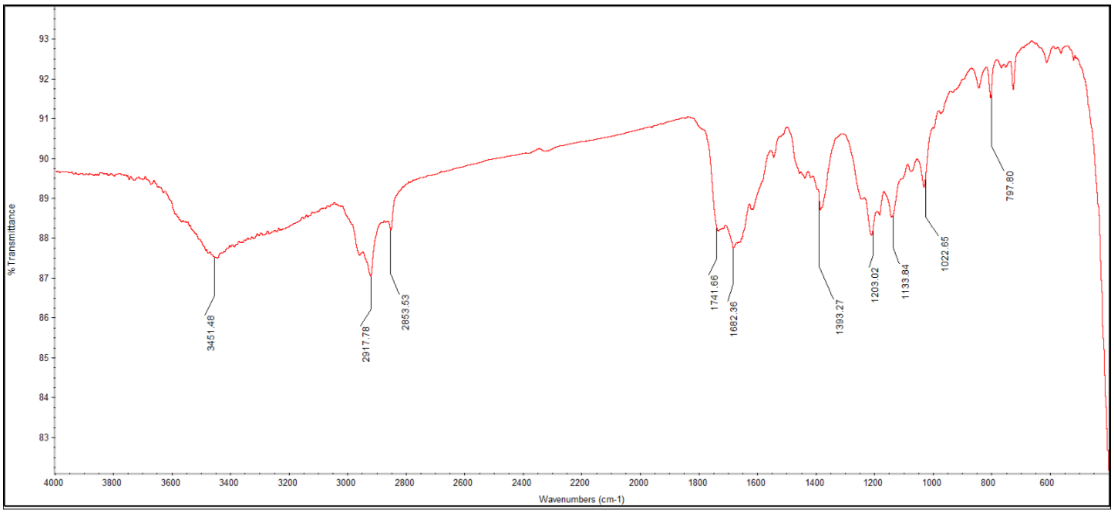

Fig. S7. IR spectrum (KBr disc) of 2α-hydroxy-7-oxo-pimara-8(9),15-diene (**1**).

Single Mass Analysis
Tolerance = 5.0 PPM / DBE: min = -1.5, max = 50.0
Element prediction: Off
Number of isotope peaks used for i-FIT = 3
Monoisotopic Mass, Even Electron Ions
109 formula(e) evaluated with 1 results within limits (up to 50 closest results for each mass)
Elements Used:

Mass	Calc. Mass	mDa	PPM	DBE	Formula	i-FIT	i-FIT Norm	Fit Conf %	C	H	N	O
303.2326	303.2324	0.2	0.7	5.5	C20 H31 O2	215.7	n/a	n/a	20	31		2

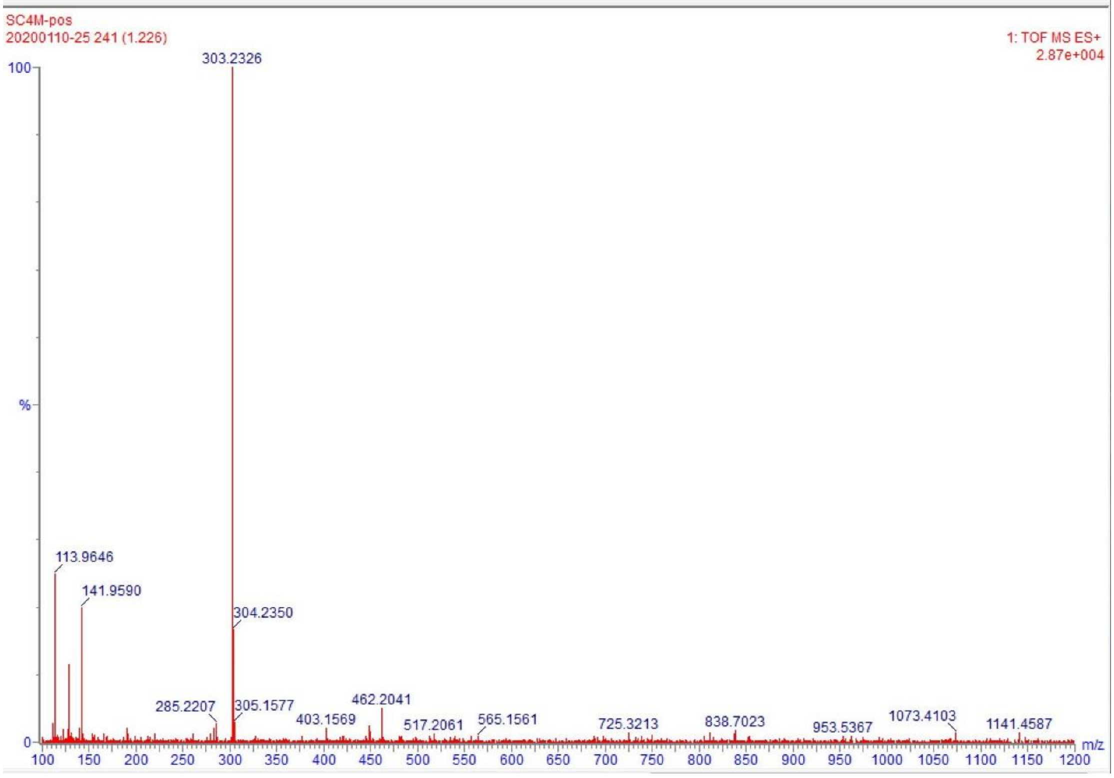

Fig. S8. HR-ESIMS spectrum of 2α-hydroxy-7-oxo-pimara-8(9),15-diene (**1**) in CH_3OH.

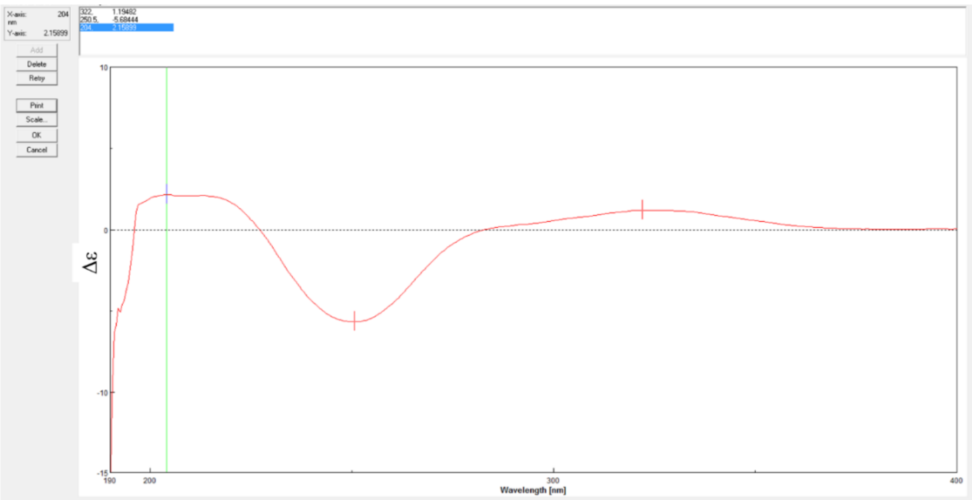

Fig. S9. ECD spectrum of 2α-hydroxy-7-oxo-pimara-8(9),15-diene (**1**) in CH_3OH.

2019-12-18 12:09:45 Page 1 of 1

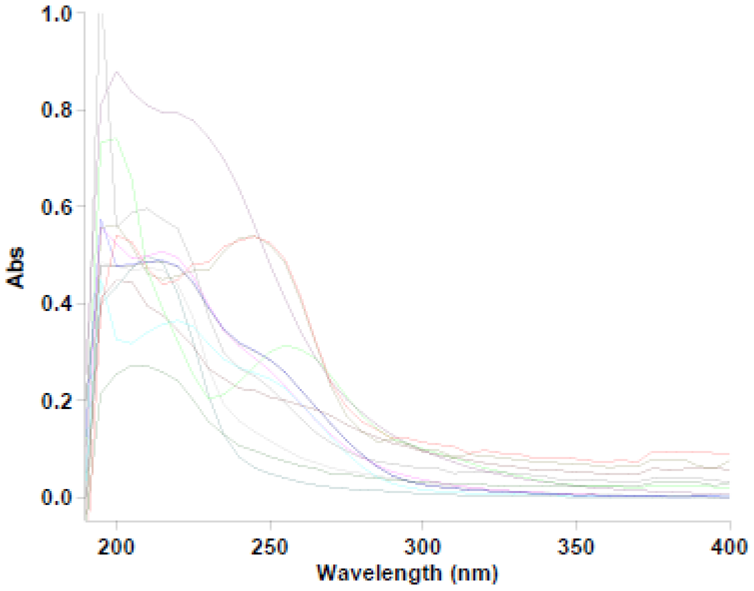

Scan Analysis Report

Report Time : 星期三 18 十二月 12:09:34 PM 2019
Method:
Batch: D:\data\ycyang\SC4M.DSW
Software version: 3.00(339)
Operator:

Sample Name: SC4M
Collection Time 2019-12-18 12:09:37

Peak Table
Peak Style Peaks
Peak Threshold 0.0100
Range 400.0nm to 190.0nm

Wavelength (nm)	Abs
245.0	0.538
200.0	0.541

Fig. S10. UV spectrum of 2α-hydroxy-7-oxo-pimara-8(9),15-diene (**1**) in CH_3OH.

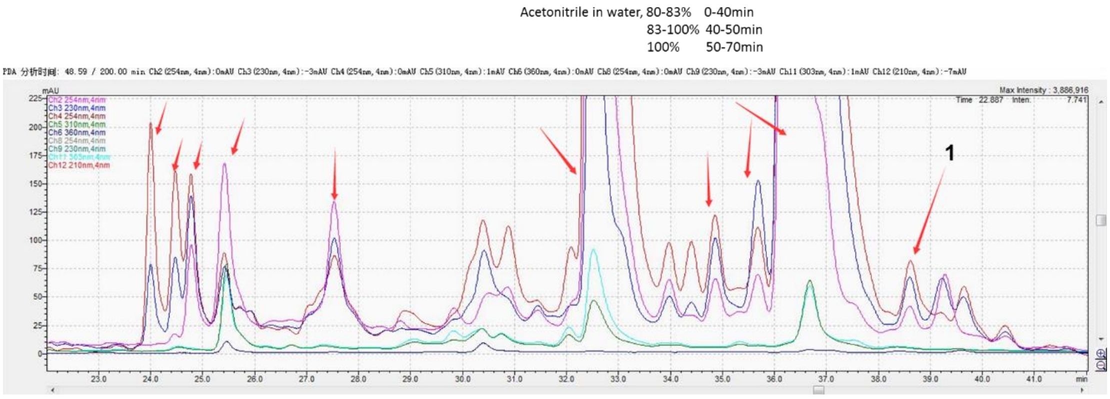

Fig. S11. Preparative HPLC chromatogram for 2α-hydroxy-7-oxo-pimara-8(9),15-diene (**1**).

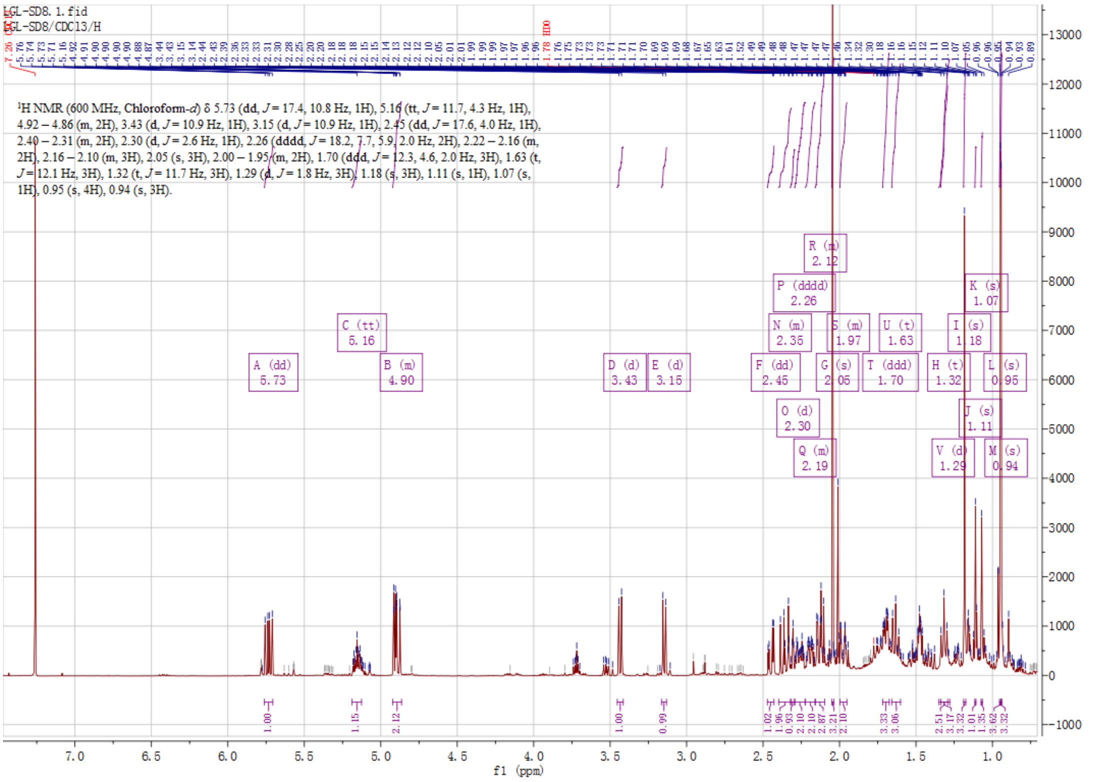

Fig. S12. ^1H NMR (600 MHz, $CDCl_3$) spectrum of 19-hydroxy-2α-acetoxy-7-oxo-pimara-8(9),15-diene (**2**).

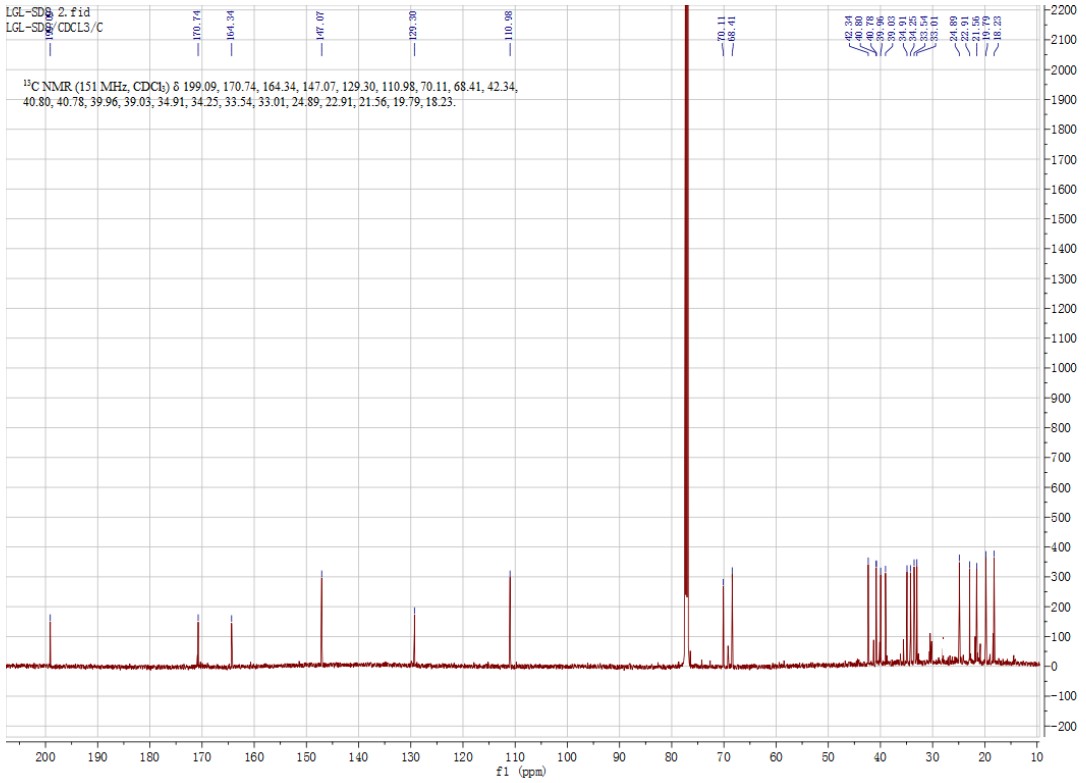

Fig. S13. ^{13}C NMR (150 MHz, $CDCl_3$) spectrum of 19-hydroxy-2α-acetoxy-7-oxo-pimara-8(9),15-diene (**2**).

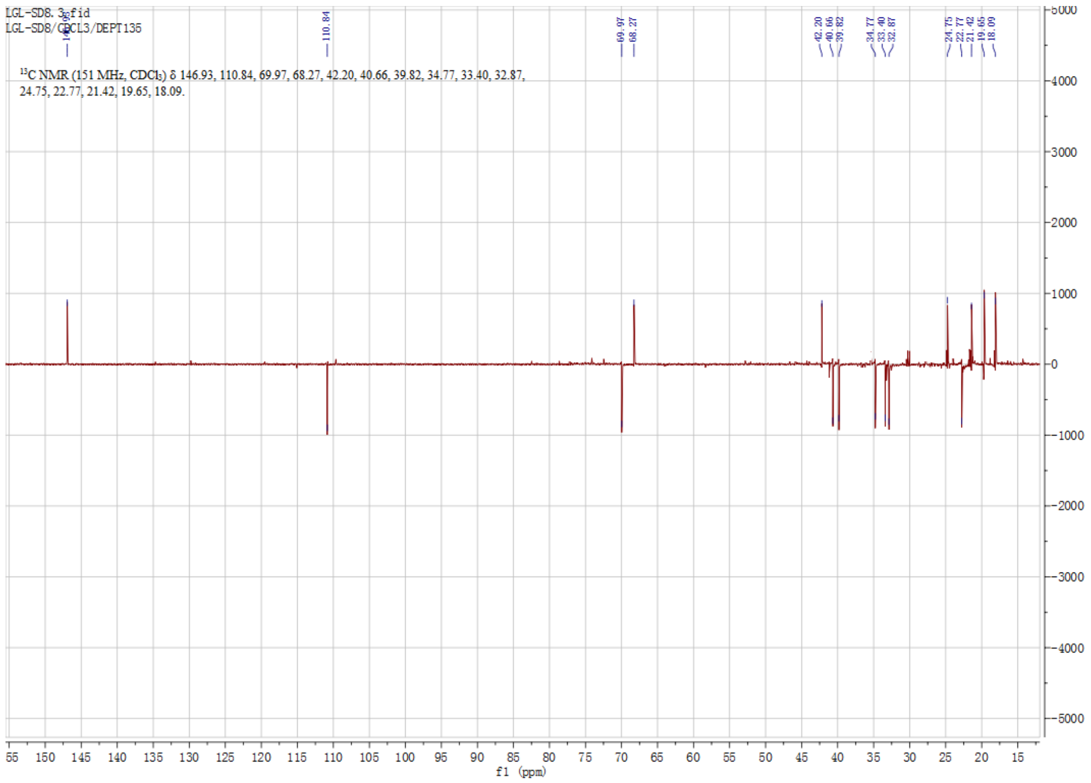

Fig. S14. DEPT-135 spectrum of 19-hydroxy-2α-acetoxy-7-oxo-pimara-8(9),15-diene (**2**).

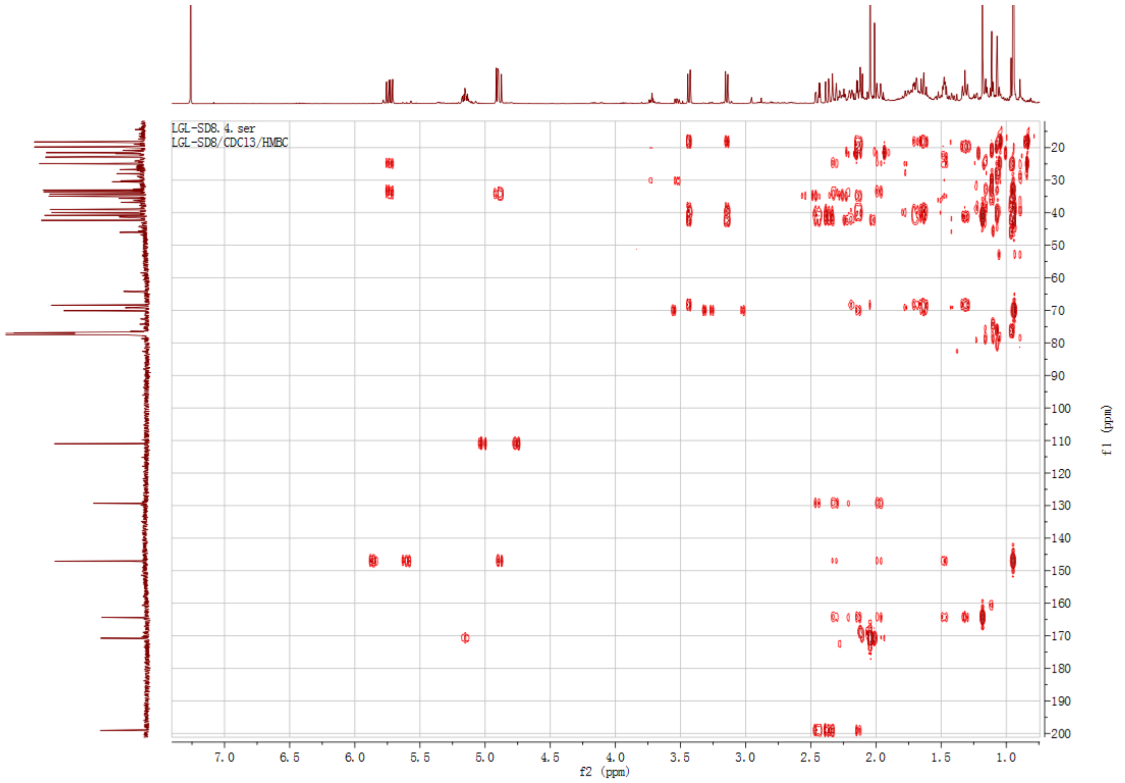

Fig. S15. HMBC spectrum of 19-hydroxy-2α-acetoxy-7-oxo-pimara-8(9),15-diene (**2**).

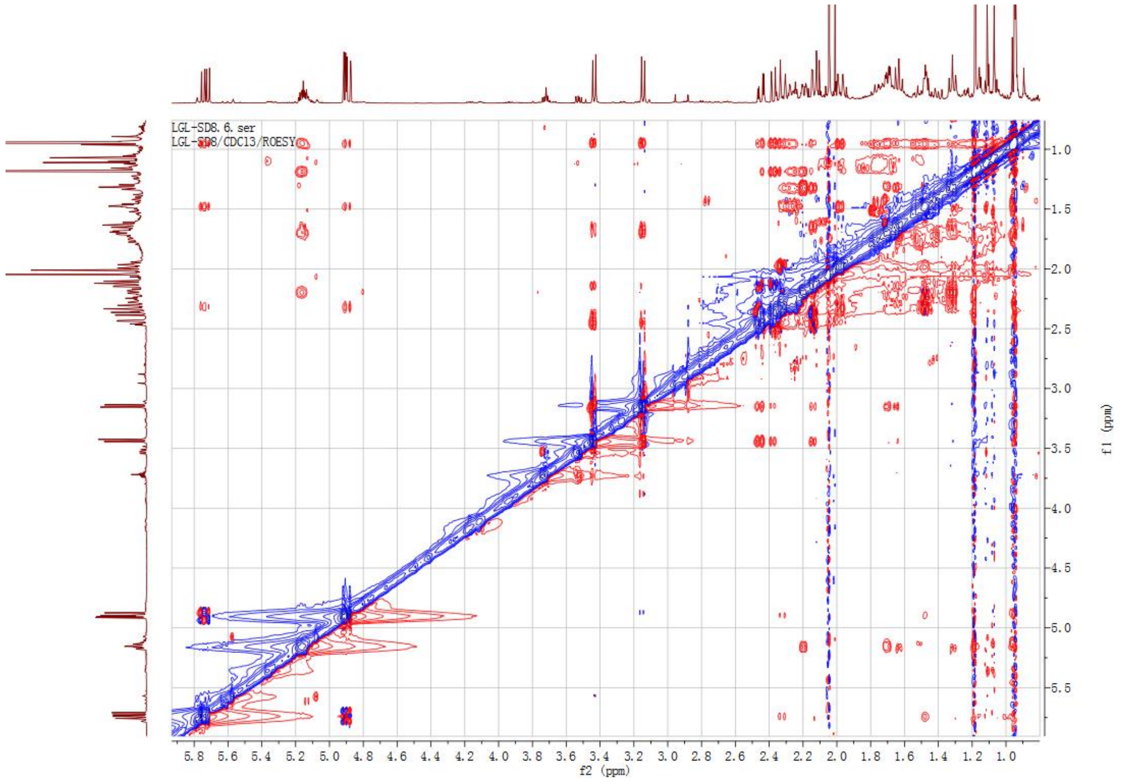

Fig. S16. ROESY spectrum of 19-hydroxy-2α-acetoxy-7-oxo-pimara-8(9),15-diene (**2**).

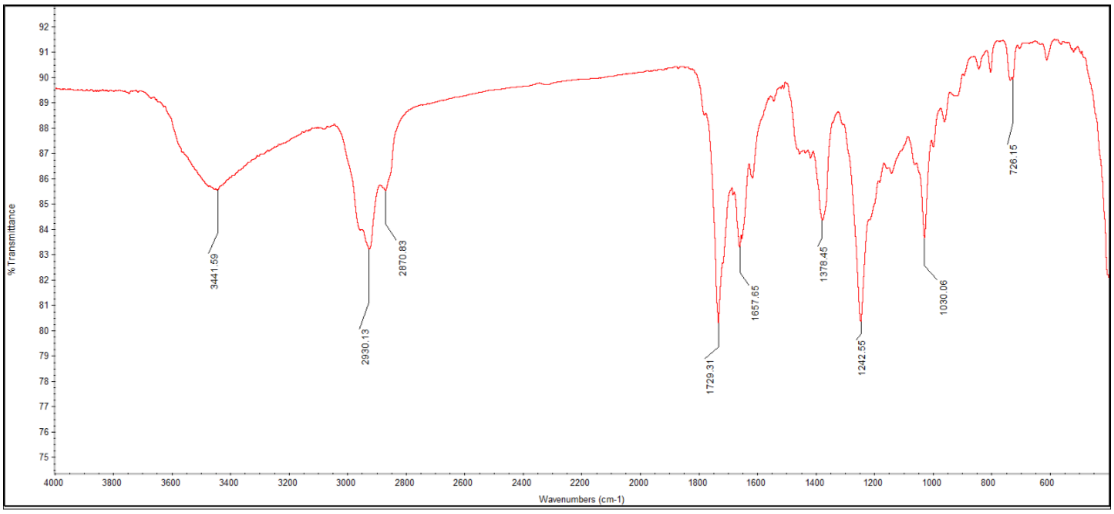

Fig. S17. IR spectrum (KBr disc) of 19-hydroxy-2*α*-acetoxy-7-oxo-pimara-8(9),15-diene (**2**).

Single Mass Analysis
Tolerance = 5.0 PPM / DBE: min = -1.5, max = 50.0
Element prediction: Off
Number of isotope peaks used for i-FIT = 3
Monoisotopic Mass, Even Electron Ions
141 formula(e) evaluated with 1 results within limits (up to 50 closest results for each mass)
Elements Used:

Mass	Calc. Mass	mDa	PPM	DBE	Formula	i-FIT	i-FIT Norm	Fit Conf %	C	H	N	O
361.2374	361.2379	-0.5	-1.4	6.5	C22 H33 O4	369.8	n/a	n/a	22	33		4

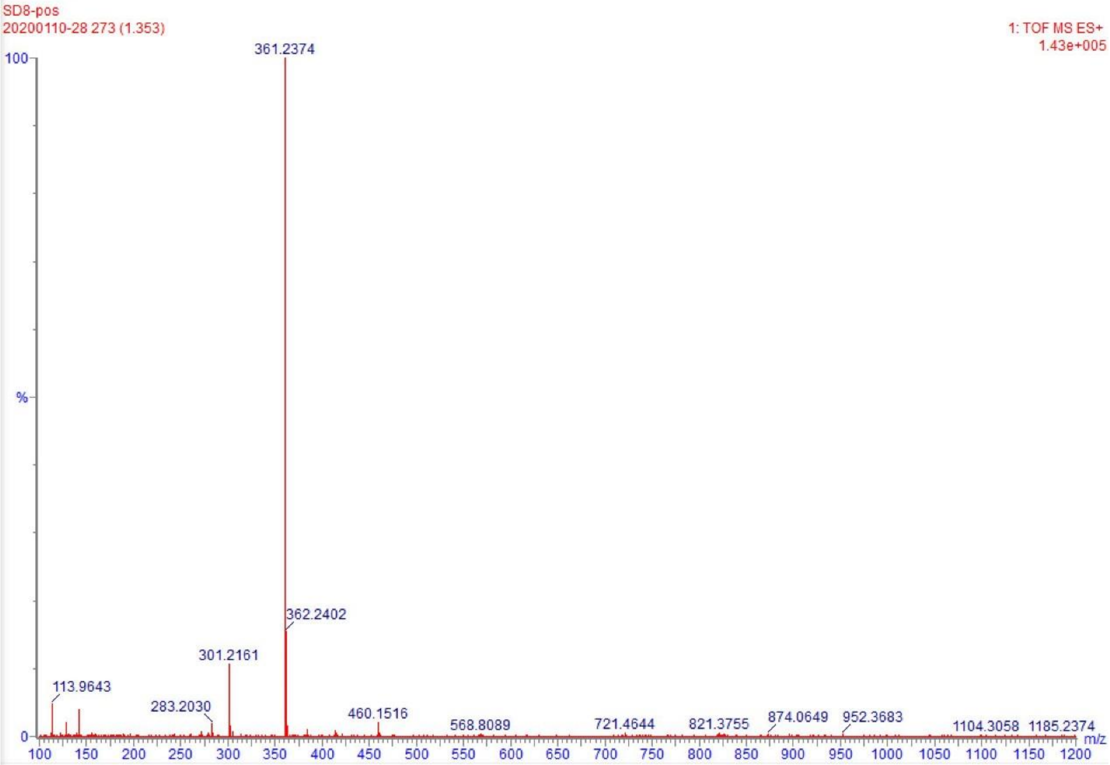

Fig. S18. HR-ESIMS spectrum of 19-hydroxy-2*α*-acetoxy-7-oxo-pimara-8(9),15-diene (**2**) in CH_3OH.

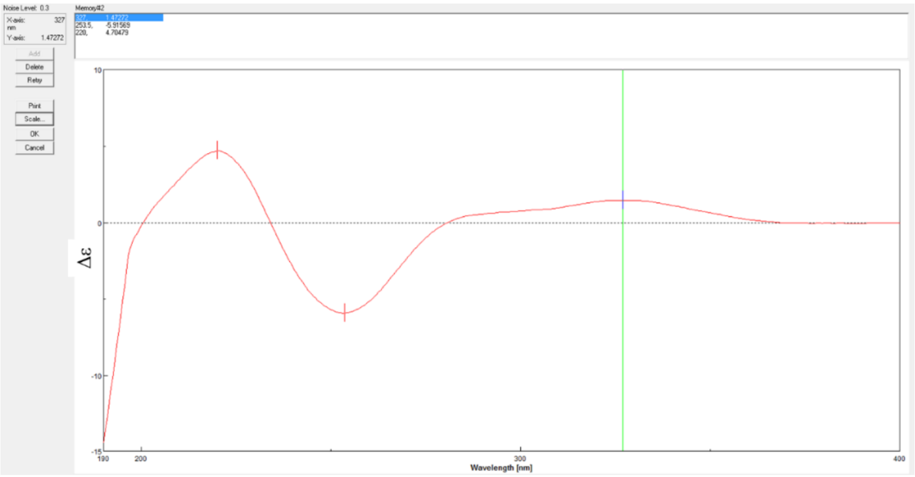

Fig. S19. ECD spectrum of 19-hydroxy-2*α*-acetoxy-7-oxo-pimara-8(9),15-diene (**2**) in CH_3OH.

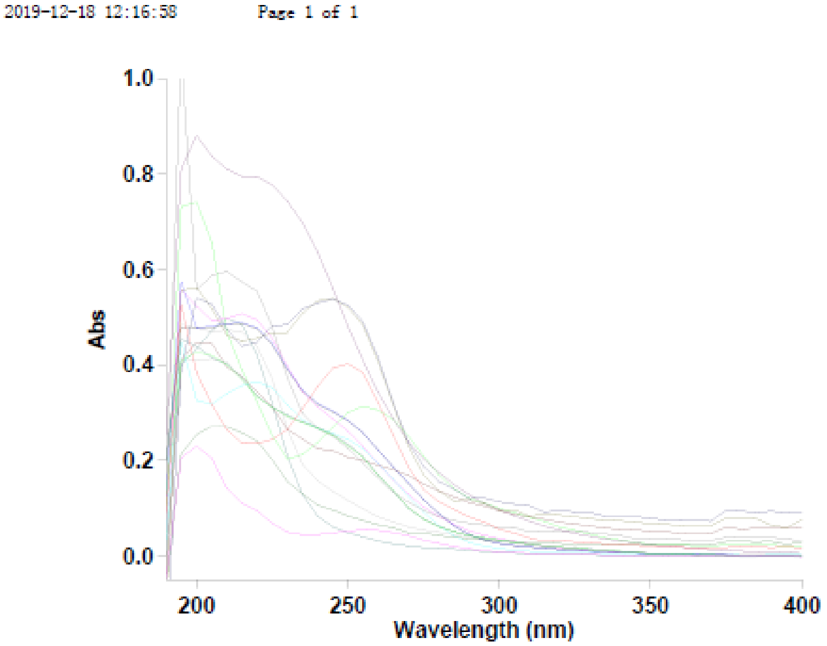

Scan Analysis Report

Report Time : 星期三 18 十二月 12:16:49 PM 2019
Method:
Batch: D:\data\ycyang\SD8.DSW
Software version: 3.00(339)
Operator:

Sample Name: SD8

Collection Time	2019-12-18 12:16:51

Peak Table

Peak Style	Peaks
Peak Threshold	0.0100
Range	400.0nm to 190.0nm

Wavelength (nm)	Abs
250.1	0.402
194.9	0.527

Fig. S20. UV spectrum of 19-hydroxy-2*α*-acetoxy-7-oxo-pimara-8(9),15-diene (**2**) in CH_3OH.

Acetonitrile in water, 80-100% 0-50min
100% 50-70min

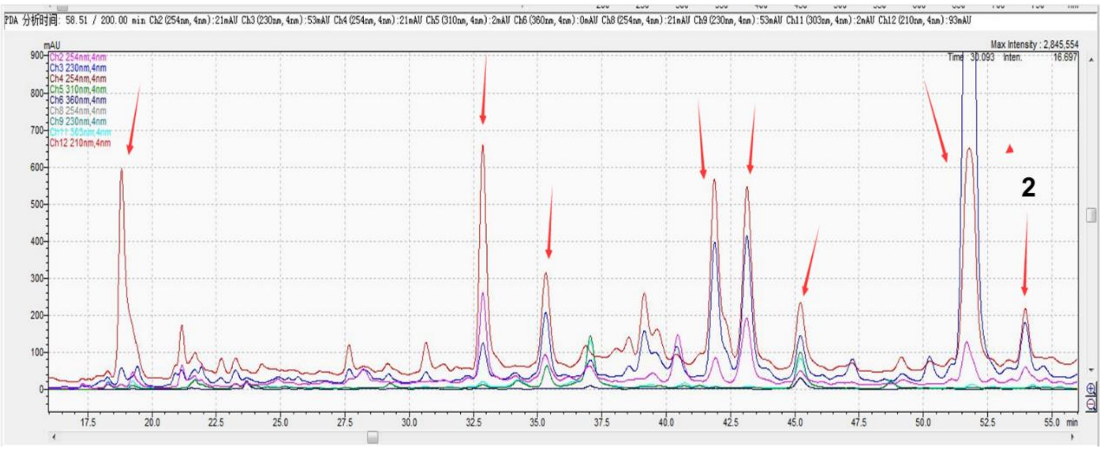

Fig. S21. Preparative HPLC chromatogram for 19-hydroxy-2α-acetoxy-7-oxo-pimara-8(9),15-diene (**2**).
